# KPNA2-Associated Immune Analyses Highlight the Dysregulation and Prognostic Effects of GRB2, NRAS, and Their RNA-Binding Proteins in Hepatocellular Carcinoma

**DOI:** 10.3389/fgene.2020.593273

**Published:** 2020-10-26

**Authors:** Xiuzhi Zhang, Jialing Zhang, Fenglan Gao, Shasha Fan, Liping Dai, Jinzhong Zhang

**Affiliations:** ^1^Department of Pathology, Henan Medical College, Zhengzhou, China; ^2^Oncology Department, The First Affiliated Hospital of Hunan Normal University, Hunan Provincial People’s Hospital, Changsha, China; ^3^Key Laboratory of Study and Discovery of Small Targeted Molecules of Hunan Province, Hunan Normal University, Changsha, China; ^4^Henan Institute of Medical and Pharmaceutical Sciences, Zhengzhou University, Zhengzhou, China

**Keywords:** hepatocellular carcinoma, immune infiltration, KPNA2, GRB2, NRAS, prognosis, RNA-binding protein

## Abstract

Karyopherin α2 (KPNA2) was reported to be overexpressed and have unfavorable prognostic effects in many malignancies including hepatocellular carcinoma (HCC). Although its contributions to inflammatory response were reported in many studies, its specific associations with immune infiltrations and immune pathways during cancer progression were unclear. Here, we aimed to identify new markers for HCC diagnosis and prognosis through KPNA2-associated immune analyses. RNA-seq expression data of HCC datasets were downloaded from The Cancer Genome Atlas and International Cancer Genome Consortium. The gene expressions were counts per million normalized. The infiltrations of 24 kinds of immune cells in the samples were evaluated with ImmuCellAI (Immune Cell Abundance Identifier). The Spearman correlations of the immune infiltrations with KPNA2 expression were investigated, and the specific positive correlation of B-cell infiltration with KPNA2 expression in HCC tumors was identified. Fifteen genes in KEGG (Kyoto Encyclopedia of Genes and Genomes) B-cell receptor signaling pathway presented significant correlations with KPNA2 expression in HCC. Among them, GRB2 and NRAS were indicated to be independent unfavorable prognostic factors for HCC overall survival. Clinical Proteomic Tumor Analysis Consortium HCC dataset was investigated to validate the results at protein level. The upregulation and unfavorable prognostic effects of KPNA2 and GRB2 were confirmed, whereas, unlike its mRNA form, NRAS protein was presented to be downregulated and have favorable prognostic effects. Through receiver operating characteristic curve analysis, the diagnostic potential of the three proteins was shown. The RNA-binding proteins (RBPs) of KPNA2, NRAS, and GRB2, downloaded *via* The Encyclopedia of RNA Interactomes, were investigated for their clinical significance in HCC at protein level. An eight-RBP signature with independent prognostic value and dysregulations in HCC was identified. All the RBPs were significantly correlated with MKI67 expression and at least one of KPNA2, GRB2, and NRAS at protein level in HCC, indicating their roles in HCC progression and the regulation of the three proteins. We concluded that KPNA2, GRB2, NRAS, and their RBPs might have coordinating roles in HCC immunoregulation and progression. They might be new markers for HCC diagnosis and prognosis predication and new targets for HCC immunotherapy.

## Introduction

Liver cancer is the seventh most common cancer and the third leading cause of cancer death worldwide (Bray et al., 2018). Hepatocellular carcinoma (HCC) is the predominant type of primary liver malignancies, with a 5-year overall survival (OS) rate of less than 20%, mainly due to its late diagnosis. Although great advancement has been made in HCC screening and treatment during the last few decades, the effectiveness still remains unsatisfactory. It was reported that for patients with early-stage HCC, the 5-year survival rate was greater than 70% (Siegel et al., 2018), much better than that of late-stage patients, calling for effective markers of its early diagnosis and prognostic predication.

Karyopherin α2 (KPNA2), also named importin α1, is a member of karyopherin α family and plays crucial roles in nucleocytoplasmic transport (Goldfarb et al., 2004). In rheumatoid arthritis, KPNA2 was shown to be a trigger of interleukin 6 (IL-6) secretion, colocalized with T cells and neutrophils, and could be upregulated *via* tumor necrosis factor *α* stimulation (Liu et al., 2015). It was also demonstrated to play crucial roles in the negative regulation of regulatory T cells (Tregs) differentiation through its interaction and translocation of proinflammatory molecule NLRP3 (Park et al., 2019). In a study of vaccinia virus (VACV), the positive effects of KPNA2 on immunoregulation was shown in the induction of VACV specific CD8^+^ T-cell memory *via* its interaction with p65 (Pallett et al., 2019). In another study, KPNA2 downregulation was reported to be associated with enterovirus 71-induced innate immune response (Peng et al., 2018), indicating its negative immunoregulatory roles.

In recent years, The dysregulation and/or prognostic roles of KPNA2 were reported in many tumors including esophageal squamous cell carcinoma (Song et al., 2019), breast cancer (Groheux et al., 2018; Wang et al., 2019a), myeloma (Kriegsmann et al., 2019; Tachita et al., 2020), clear cell renal cell carcinoma (Müller et al., 2019), oral cancer (Wang et al., 2018), bladder cancer (Jeong et al., 2017), and colorectal cancer (Ostasiewicz et al., 2016; Jeong et al., 2017). The immune-related roles of KPNA2 were also shown in tumors. In colorectal cancer cells, upregulated KPNA2 was demonstrated to be associated with immunogenic cell death (Song et al., 2016). In breast cancer, KPNA2 knockdown could suppress the inflammatory responses and malignant progression of the tumor cells induced by IL-6 (Duan et al., 2020). In HCC, KPNA2 overexpression and its prognostic effects were also reported in several studies (Jiang et al., 2014; Yang et al., 2017b; Chen et al., 2019; Guo et al., 2019; Liu et al., 2019; Yue et al., 2019; Yu et al., 2020). In fact, its tumor-promoting activities were also shown (Gao et al., 2018; Lin et al., 2018; Zan et al., 2019) in HCC. However, although the correlations between KPNA2 expression and immune infiltrations were investigated in a recent study (Hua et al., 2020), its associations with immune response in HCC were not clearly illustrated. Furthermore, with only one dataset and only six kinds of immune cells included, the reliability of the results was limited.

Considering the close relationship between inflammation and HCC (El-Serag et al., 2008), we speculated that KPNA2 upregulation might participate in or associate with specific immune pathways during HCC progression. In this study, we evaluated the correlations between KPNA2 expression and infiltrations of 24 kinds of immune cells in HCC tumors and normal liver tissues from The Cancer Genome Atlas (TCGA) and International Cancer Genome Consortium (ICGC) HCC datasets to identify the specific correlations between KPNA2 expression and immune infiltration in HCC tumors in contrast to those in normal liver tissues. The Kyoto Encyclopedia of Genes and Genomes (KEGG) B-cell receptor (BCR) signaling pathway genes were analyzed and validated for their correlations with KPA2 expression and their independent prognostic effects in HCC. Considering the crucial roles of RNA-binding proteins (RBPs) in RNA splicing, RNA translation, and RNA degradation (Mohibi et al., 2019), the RBPs of KPNA2 and its correlated BCR signaling pathway genes with prognostic values were further investigated to find their roles in the dysregulations of the identified genes/proteins and their diagnostic value and prognostic effects in HCC. The results here might provide new clues for the roles of KPNA2 in immunoregulation, new markers for HCC early diagnosis and prognostic predication, and new targets for HCC immunotherapy.

## Materials and Methods

### Data Collection

RNA-seq data of HCC tumors and normal liver tissues in HCC datasets with the clinical information were downloaded from Genomic Data Commons data portal (TCGA-LIHC dataset, called TCGA-HCC in this study) and ICGC database (ICGC-HCC dataset). There were 371 primary tumors and 50 normal liver tissues from 371 HCC patients in the TCGA-HCC dataset and 223 primary tumors and 202 normal tissues from 223 HCC patients in the ICGC-HCC dataset. The clinical features of the patients are shown in Table 1. The gene expression read count data of the two datasets were counts per million (CPM) transformed for normalization for further analyses.

**Table 1:** Clinical features of the patients in the TCGA-HCC dataset (*n* = 371) and ICGC-HCC (*n* = 223) dataset.

Variables	TCGA-HCC (*n* = 371), *n* (%)	ICGC-HCC (*n* = 223), *n* (%)
Gender		
Male	250 (67.4%)	164 (73.5%)
Female	121 (32.6%)	59 (26.5%)
Age		
≤60	177 (47.7%)	47 (21.1%)
>60	193 (52.0%)	176 (78.9%)
NA	1 (0.3%)	0 (0%)
TNM stage		
I	171 (46.1%)	36 (15.7%)
II	86 (23.2%)	102 (45.7%)
III	85 (22.9%)	67 (30.5%)
IV	5 (1.3%)	18 (8.1%)
NA	24 (6.5%)	0 (0%)
Grade		
G1	55 (14.8%)	20 (9.0%)
G2	177 (47.7%)	128 (57.4%)
G3	122 (32.9%)	56 (45.5%)
G4	12 (3.2%)	1 (0.4%)
NA	5 (1.3%)	18 (8.1%)
Survival status		
Alive	240 (64.7%)	181 (81.2%)
Dead	130 (35.0%)	42 (18.8%)
NA	1 (0.3%)	0 (0%)

HCC, hepatocellular carcinoma; ICGC, International Cancer Genome Consortium; NA, not available; TCGA, The Cancer Genome Atlas.

### Correlation Analyses Between KPNA2 Expression and Immune Cell Infiltrations and the Investigation of Prognostic Roles of B-Cell Infiltration in HCC

With their gene expression data, the infiltrations of 24 kinds of immune cells including 18 T-cell subsets [CD4^+^, CD8^+^, CD4^+^ naive, CD8^+^ naive, central memory T (Tcm), effector memory T, Tr1, iTreg, nTreg, T_H_1, T_H_2, T_H_17, Tfh, cytotoxic T, MAIT, exhausted T (Tex), gamma delta T (γδ T), and natural killer T (NKT) cells] and six other important immune cells [B cells, macrophages, monocytes, neutrophils, dendritic cell (DC), and NK cells] in the HCC tumors and normal liver tissues from the TCGA-HCC and ICGC-HCC datasets were evaluated with ImmuCellAI (Immune Cell Abundance Identifier; Miao et al., 2020). The correlations between KPNA2 expression and the immune cell infiltrations of the HCC tumors and normal liver tissues were evaluated individually. Spearman correlation analysis was used, and a |correlation coefficient| > 0.15 with *p* < 0.01 was considered as statistically significant.

Kaplan-Meier survival analysis was used to evaluate the prognostic effects of B-cell infiltration on HCC OS with a cutoff value of B-cell infiltration identified from survminer package in R.^1^ To investigate the independent prognostic effects of B-cell infiltration HCC OS, multivariable Cox regression analysis with ezcox package (Wang et al., 2019b) in R was performed and the gender-, age-, and stage-corrected prognostic effects of B-cell infiltration were evaluated in the TCGA-HCC and ICGC-HCC datasets individually. For survival analyses, only the patients with survival time >0 were included.

### Identification of KPNA2-Correlated BCR Signaling Pathway Genes

KEGG BCR signaling pathway (hsa04662) genes (*n* = 75) were investigated through KEGG database.^2^ Their expressions were analyzed for their correlations with KPNA2 expression in HCC samples form TCGA-HCC and ICGC-HCC datasets. Spearman correlation analysis was used, and a |correlation coefficient| > 0.15 with *p* < 0.01 was considered as statistically significant.

### Differential Expression Analyses and Prognostic Effects Evaluation of the KPNA2-Correlated BCR Signaling Pathway Genes in HCC

The BCR signaling pathway genes with significant correlations with KPNA2 expression were selected, and their expression profiles and prognostic roles were investigated in the TCGA-HCC and ICGC-HCC datasets. Gene expression comparisons between HCC tumors and normal liver tissues were performed with Wilcoxon test. Univariable Cox regression analysis was performed to evaluate the prognostic effects of the genes. The independent prognostic values were investigated through multivariable Cox regulation analysis with ezcox package (Wang et al., 2019b) in R software with the gene expressions as covariates and gender, age, and stage as controls. Only the BCR signaling pathway genes with prognostic effects independent of gender, age, and stage were selected for further analyses. For the analyses, *p* < 0.05 was considered significant.

### Validation of the Dysregulation and Prognostic Effects of KPNA2, GRB2, and NRAS at Protein Level

To validate the results above, the HCC proteomic data of a Chinese cohort from Clinical Proteomic Tumor Analysis Consortium (CPTAC; Gao et al., 2019) was investigated. The tumor samples (*n* = 159) and their paired liver tissues (*n* = 159) from 159 HCC patients were included. The clinical information of the patients is shown in Supplementary Table S1. The expressional differences of the proteins were evaluated with paired-samples Wilcoxon test. The diagnostic power of the proteins was evaluated through receiver operating characteristic (ROC) curve analysis with pROC package (Robin et al., 2011) in R. The area under the curve (AUC) and the sensitivity and specificity at the cutoff point with the biggest Youden index (sensitivity + specificity − 1) were evaluated. The correlations of the protein expressions were investigated through Spearman correlation analysis. The prognostic effects of KPNA2, GRB2, and NRAS on HCC OS were investigated through Kaplan-Meier survival analysis for which survminer package in R was used to get the cutoff value for grouping the patients.^3^ Furthermore, ezcox package (Wang et al., 2019b) in R was used to evaluate the age-, gender-, and tumor size-corrected prognostic effects of the proteins. For all the analyses, *p* < 0.05 as statistically significant.

### Exploration of the Potential Roles of KPNA2, GRB2, and NRAS in HCC Proliferation

To explore the associations of KPNA2, GRB2, and NRAS with HCC proliferation, these correlations with cell proliferation MKI67 (Gerdes et al., 1983; Scholzen and Gerdes, 2000; Booth et al., 2014) were evaluated in HCC tumor samples through Spearman correlation analysis, and *p* < 0.05 was considered statistically significant.

### Further Investigation of the Prognostic Effects and Dysregulation of RBPs of KPNA2, GRB2, and NRAS in HCC

RBP binding at specific target sites could impact the expression of functionally coordinated sets of mRNAs and regulate their function in the cell (Sternburg and Karginov, 2020). Here, through The Encyclopedia of RNA Interactomes (ENCORI),^4^ the RBPs of KPNA2 and its correlated BCR signaling pathway genes (GRB2 and NRAS) were also investigated. The gender-, age-, and tumor size-corrected prognostic effects of the RBPs were analyzed through multivariable Cox regression analysis with ezcox package (Wang et al., 2019b) in R software. The RBPs with prognostic effects independent of gender, age, and tumor size, as well as KPNA2, GRB2, and NRAS, were then applied to the least absolute shrinkage and selection operator (LASSO) Cox regression to get an effective risk model for the prognostic predication of HCC. The risk score of each patient was evaluated with the formula as follows:

$$\mathrm{risk score}=\overset{n}{\underset{i=1}{\sum }}\mathrm{coefficient}\left(i\right)*\mathrm{expression}\left(i\right)$$

where *n* is the number of selected proteins, coefficient(*i*) is the coefficient of the protein *i*, and expression(*i*) is the expression level of protein *i*. The accuracy of the model in the survival status predication of HCC patients was investigated through ROC curve analysis, and the AUC was evaluated. Then, the HCC patients were than divided into high‐ and low-risk groups with the median risk score as the threshold. Through Kaplan-Meier survival analysis, the survival differences between high‐ and low-risk patients were visualized. The independent prognostic value of the model was also evaluated through Kaplan-Meier survival analysis in the high‐ and low-risk patients of different tumor size groups, different gender groups, and different age groups individually.

For the RBPs in the model above, their expressional differences between HCC tumors and their paired normal liver tissues were investigated through paired-samples Wilcoxon test, and their diagnostic value was investigated through ROC curve analysis with pROC package (Robin et al., 2011) in R. To further investigate their potential roles in the dysregulation of KPNA2, GRB2, and NRAS, their Spearman correlations in HCC were also evaluated. Furthermore, to investigate their associations with HCC proliferation, their correlations with proliferation marker MKI67 were evaluated through spearman correlation analyses. For these analyses, *p* < 0.05 was considered significant.

## Results

### Positive Correlation of B-Cell Infiltration With KPNA2 Expression and Its Prognostic Roles in HCC

**Figure 1:**
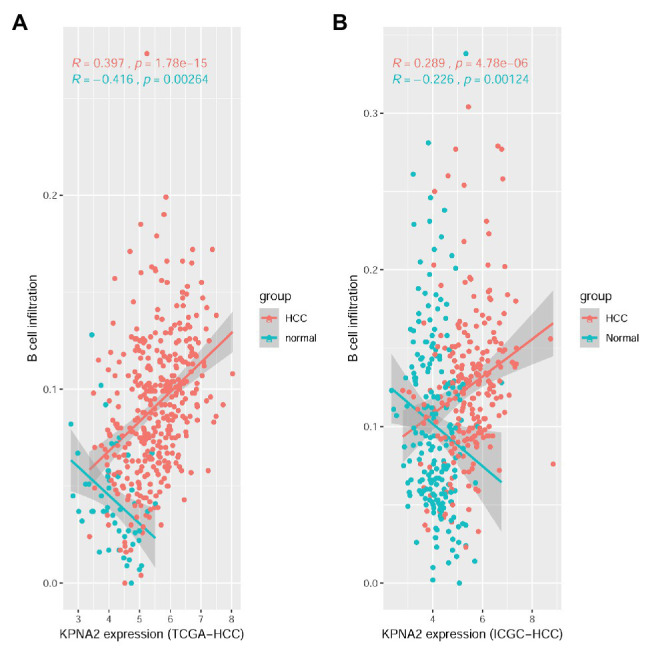
Spearman correlation analysis between KPNA2 expression and B-cell immune infiltrations in HCC. **(A)** KPNA2 expression was positively correlated with B-cell immune infiltration in HCC tumors while negatively correlated with B-cell infiltration in normal liver tissues in the TCGA-HCC dataset. **(B)** KPNA2 expression was positively correlated with B-cell immune infiltration in HCC tumors while negatively correlated with B-cell infiltration in normal liver tissues in the ICGC-HCC dataset. Spearman correlation analysis was used. |*R|* > 0.15 with *p* < 0.01 was considered statistically significant. *R*, correlation coefficient.

For the TCGA-HCC dataset, as shown in Supplementary Figure S1, 10 of the immune cell infiltrations were shown to be negatively (*R* < −0.15 and *p* < 0.01: CD4^+^ naive, T_H_17, Tfh, MAIT, NK, and CD4^+^ T infiltrations) or positively correlated (*R* > 0.15 and *p* < 0.01: Tex, nTreg, DC, and B-cell infiltrations) with KPNA2 expression in HCC tumors in the TCGA-HCC datasets. In the normal liver tissues of the TCGA-HCC dataset, the negative correlations (*R* < −0.15 and *p* < 0.01) of CD4^+^ naive and B-cell and the positive correlation (*R* > 0.15 and *p* < 0.01) of DC infiltration with KPNA2 expression were shown. Interestingly, B-cell infiltration was shown to be positively correlated (*R* = 0.397, *p* < 0.01) with KPNA2 expression in HCC tumors while negatively correlated (*R* = −0.416, *p* < 0.01) with KPNA2 expression in the normal liver tissues (Figure 1A).

For the ICGC-HCC dataset, as shown in Supplementary Figure S2, six kinds of immune cells including CD4^+^ naive, Tfh, MAIT, monocyte, NK cell, and CD4^+^ T cell were shown to be significantly negatively correlated (*R* < −0.15 and *p* < 0.01), while DC and B-cell infiltrations were positively correlated (*R* > 0.15 and *p* < 0.05) with KPNA2 expression in HCC tumors. In the normal liver tissues, negative correlations (*R* < −0.15 and *p* < 0.01) of CD4^+^ naive, B cell, NK, γδ T, and CD4^+^ T infiltrations, whereas positive correlations (*R* > 0.15 and *p* < 0.01) of Tcm, DC, and macrophage infiltrations with KPNA2, were shown. Noticeably, opposite correlations of B-cell infiltration with KPNA2 expression in HCC tumors (*R* = 0.289, *p* < 0.01) and the normal liver tissues (*R* = −0.226, *p* < 0.01) were also obvious here (Figure 1B), consistent with the results in the TCGA-HCC dataset, indicating the specificity of the positive correlation between KPNA2 expression and B-cell infiltration in the HCC tumors in contrast to the normal controls.

**Figure 2:**
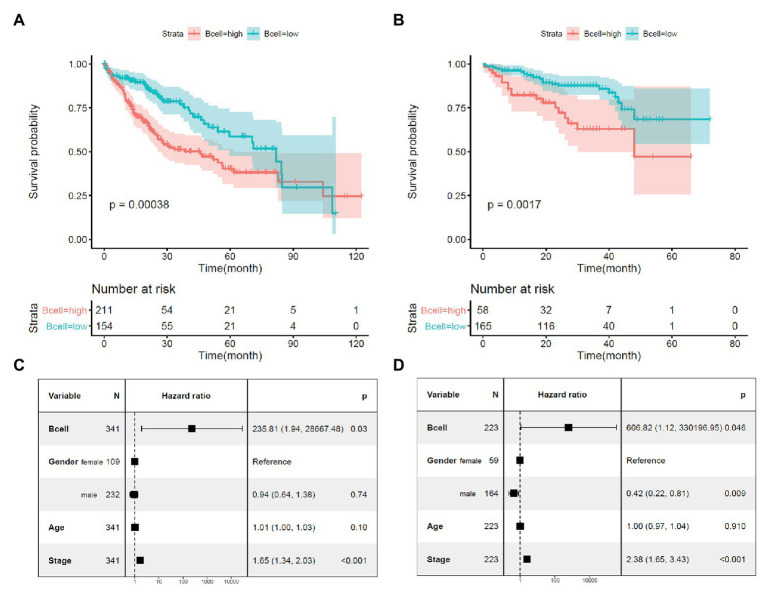
Prognostic effects of B-cell infiltration in HCC. **(A)** Significant shorter survival in the HCC patients with high B-cell infiltration than the patients with low B-cell infiltration in the TCGA-HCC dataset. **(B)** Significant shorter survival in the HCC patients with high B-cell infiltration than the patients with low B-cell infiltration in the ICGC-HCC dataset. **(C)** Prognostic effects of B-cell infiltration on HCC overall survival when adjusted for gender, age, and stage in the TCGA-HCC dataset. **(D)** Prognostic effects of B-cell infiltration on HCC overall survival when adjusted for gender, age, and stage in the ICGC-HCC dataset. Kaplan-Meier survival analysis with log-rank test and multivariable Cox regression analysis were used for evaluation of the prognostic effects of B-cell infiltration. For all the analyses, *p* < 0.05 was considered statistically significant.

Through Kaplan-Meier survival analysis (Figures 2A,B) and multivariable Cox regression analysis (Figures 2C,D), the unfavorable prognostic effects of B-cell infiltration on HCC OS were shown both in the TCGA-HCC and ICGC-HCC datasets, indicating its potential in HCC prognosis predication. BCR is a master regulator of B cells. Through BCR, the B cells recognize foreign antigen, leading to maturation of the B-cell into either a memory B-cell or an effector (plasma) B-cell (Puri et al., 2013; Skånland et al., 2020). We speculated that there might be also associations between BCR signaling pathway genes and KPNA2, and they might play important roles during HCC progression.

### Correlations Between KPNA2 Expression and BCR Signaling Pathway Genes in HCC

**Figure 3:**
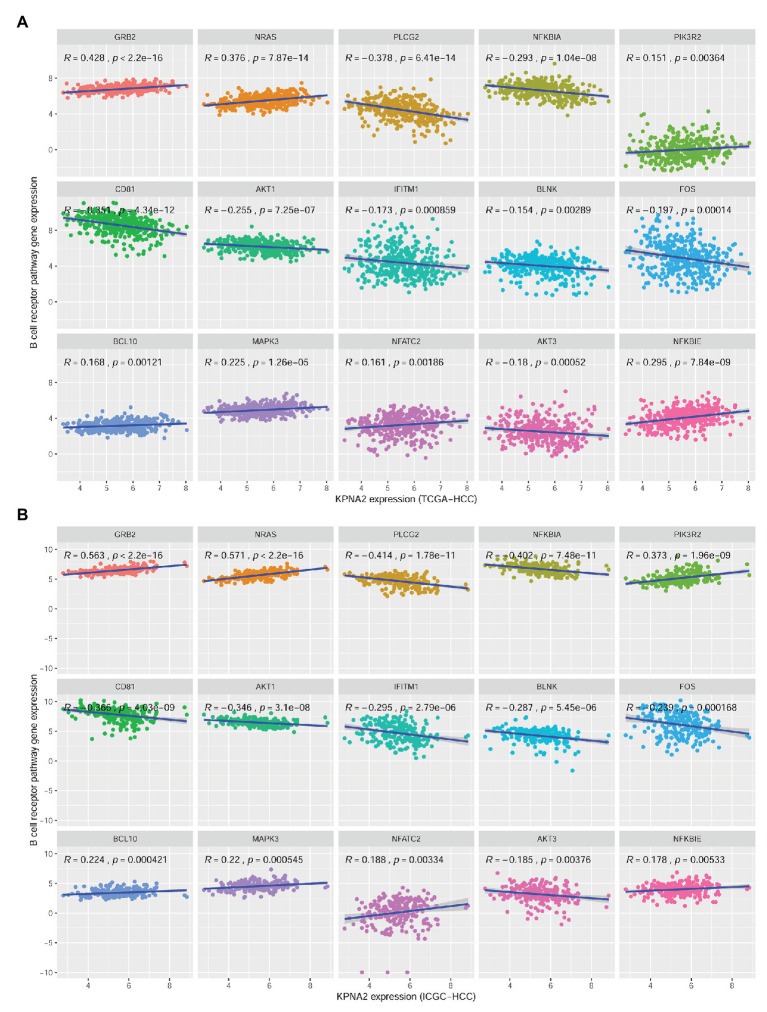
Significant correlations between BCR signaling pathway gene expressions and KPNA2 expression in HCC. **(A)** Significant positive correlations of GRB2, NRAS, NFKBIE, MAPK3, BCL10, NFATC2, and PIK3R2 and negative correlations of BLNK, IFITM1, AKT3, FOS, AKT1, NFKBIA, CD81, and PLCG2 with KPNA2 expression in HCC in the TCGA-HCC dataset. **(B)** Significant positive correlations of GRB2, NRAS, NFKBIE, MAPK3, BCL10, NFATC2, and PIK3R2 and negative correlations of BLNK, IFITM1, AKT3, FOS, AKT1, NFKBIA, CD81, and PLCG2 with KPNA2 expression in HCC in the ICGC-HCC dataset. The *x*-axis and *y*-axis represented the relative expression of KPNA2 and BCR signaling pathway genes, respectively. The gene expressions were CPM normalized and log_2_(*x* + 0.001) transformed. BCR, B-cell receptor; *R*, correlation coefficient; CPM, count per million. Spearman correlation was used, and |*R*| ≥ 0.15 with *p* < 0.01 was considered significant.

There were 27 BCR signaling pathway genes negatively or positively correlated with KPNA2 at mRNA level in HCC tumors of the TCGA-HCC and ICGC-HCC datasets individually (Supplementary Table S2). Among them, seven genes including GRB2, NRAS, NFKBIE, MAPK3, BCL10, NFATC2, and PIK3R2 were positively correlated (*R* > 0.15 and *p* < 0.01), whereas eight genes including BLNK, IFITM1, AKT3, FOS, AKT1, NFKBIA, CD81, and PLCG2 were negatively correlated (*R* < −0.15 and *p* < 0.01) with KPNA2 expression in HCC in both TCGA-HCC dataset (Figure 3A) and ICGC-HCC dataset (Figure 3B).

### Differential Expression and Prognostic Effects of KPNA2 and Its Correlated BCR Signaling Pathway Genes in HCC

**Figure 4:**
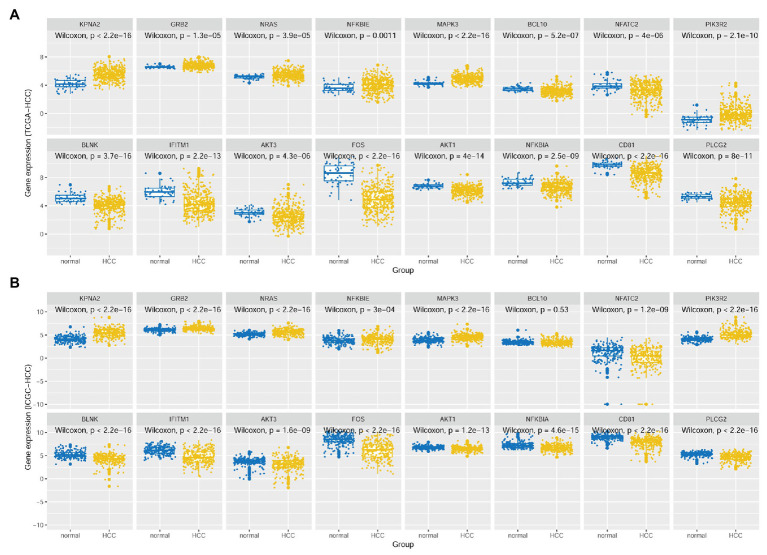
Expressional comparisons of KPNA2 and its correlated BCR signaling pathway genes between HCC tumors and normal livers. **(A)** Expressional differences of GRB2, NRAS, NFKBIE, MAPK3, BCL10, NFATC2, PIK3R2, BLNK, IFITM1, AKT3, FOS, AKT1, NFKBIA, CD81, and PLCG2 between HCC tumors and normal livers in the TCGA-HCC dataset. **(B)** Expressional differences of GRB2, NRAS, NFKBIE, MAPK3, BCL10, NFATC2, PIK3R2, BLNK, IFITM1, AKT3, FOS, AKT1, NFKBIA, CD81, and PLCG2 between HCC tumors and normal livers in the ICGC-HCC dataset. The *x*-axis and *y*-axis represented the sample groups and the relative expressions of the genes, respectively. The gene expressions were CPM normalized and log_2_(*x* + 0.001) transformed. BCR, B-cell receptor; CPM, count per million. Wilcoxon test was used for comparisons, and *p* < 0.05 was considered significant.

As shown in Figure 4, besides the overexpression of KPNA2 in HCC tumors, among the seven-KPNA2 positively correlated BCR signaling pathway genes, six genes including GRB2, NRAS, NFKBIE, MAPK3, and NFATC2 were shown to be higher expressed, whereas NFATC2 was lower expressed in the HCC tumors than the normal liver tissues (*p* < 0.05) in both TCGA-HCC dataset (Figure 4A) and ICGC-HCC dataset (Figure 4B). However, BCL10 was shown to be downregulated in the TCGA-HCC tumors (*p* < 0.05) but not statistically significant in the ICGC-HCC tumors (*p* > 0.05). For the eight-KPNA2 negatively correlated BCR signaling pathway genes, all of them were shown to be downregulated in HCC tumors comparing with the normal liver tissues in the two datasets (*p* < 0.05).

**Figure 5:**
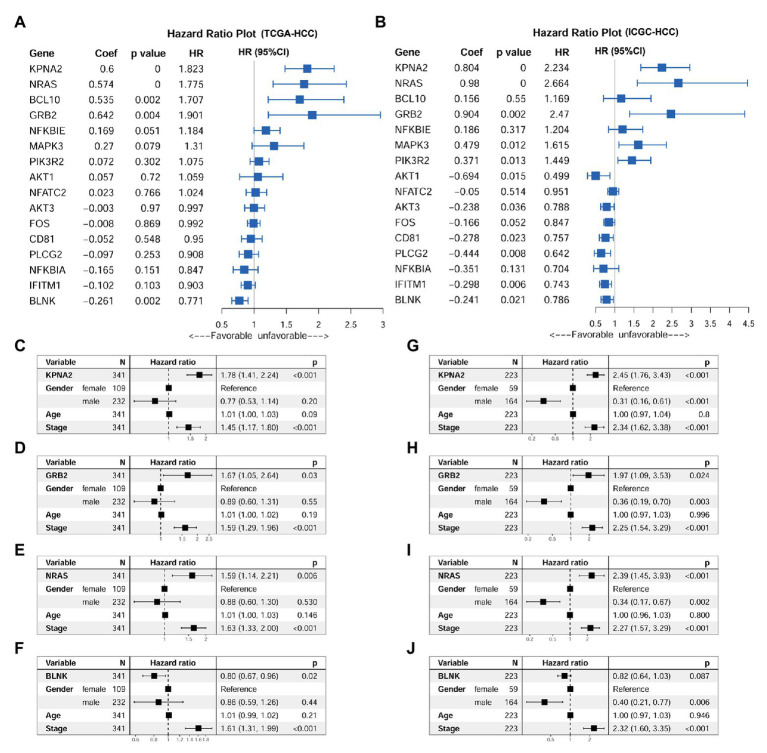
Prognostic effects of KPNA2 and its correlated BCR signaling pathway genes in HCC. **(A)** Prognostic effects of KPNA2 and its correlated BCR signaling pathway genes on overall survival of the TCGA-HCC patients through univariable Cox regression analysis. **(B)** Prognostic effects of KPNA2 and its correlated BCR signaling pathway genes on overall survival of the ICGC-HCC patients through univariable Cox regression analysis. **(C–F)** The gender-age-stage-corrected prognostic effects of KPNA2, GRB2, NRAS, and BLNK on OS of the TCGA-HCC patients, respectively. **(G–J)** The gender-age-stage–corrected prognostic effects of KPNA2, GRB2, NRAS, and BLNK on overall survival of the ICGC-HCC patients, respectively. For **(A,B)**, univariable Cox regression analysis was used. For **(C–J)**, multivariable Cox regression analysis was performed. The analyses were performed with R software, and *p* < 0.05 was considered statistically significant.

Through univariable Cox regression analysis (Figures 5A,B), besides the unfavorable prognostic effects of KPNA2 (HR_TCGA-HCC_ = 1.823, HR_ICGC-HCC_ = 2.234, *p* < 0.05), two of its positively correlated BCR signaling pathway genes (NRAS: HR_TCGA-HCC_ = 1.775, HR_ICGC-HCC_ = 2.664, *p* < 0.05; GRB2: HR_TCGA-HCC_ = 1.901, HR_ICGC-HCC_ = 2.470, *p* < 0.05) and one of its negatively correlated genes (BLNK: HR_TCGA-HCC_ = 0.771, HR_ICGC-HCC_ = 0.786, *p* < 0.05) were shown to be prognostic factors both in the TCGA-HCC patients and ICGC-HCC patients. For the other 12 genes, their prognostic effects were shown in only one of or neither of the two datasets. Then, KPNA2 and the three BCR signaling pathway genes (GRB2, NRAS, and BLNK) with consistent prognostic effects in the two datasets were selected for further investigation of their independent prognostic values. When their prognostic effects were adjusted for gender, age, and tumor stage, the unfavorable effects of KPNA2 (HR_TCGA-HCC_ = 1.78, HR_ICGC-HCC_ = 2.45, *p* < 0.05; Figures 5C,G), GRB2 (HR_TCGA-HCC_ = 1.67, HR_ICGC-HCC_ = 1.97, *p* < 0.05; Figures 5D,H), and NRAS (HR_TCGA-HCC_ = 1.59, HR_ICGC-HCC_ = 2.39, *p* < 0.05; Figures 5E,I) on HCC OS also existed in both TCGA-HCC and ICGC-HCC datasets, indicating their potential in the predication of HCC prognosis. However, although the independent favorable prognostic effect of BLNK in the TCGA-HCC (HR = 0.80, *p* < 0.05; Figure 5F) was shown, it was not so significant in the ICGC-HCC dataset (HR = 0.82, *p* > 0.05; Figure 5J).

### Validation of Dysregulations, Correlations, and Prognostic Effects of KPNA2, GRB2, and NRAS in HCC at Protein Level

**Figure 6:**
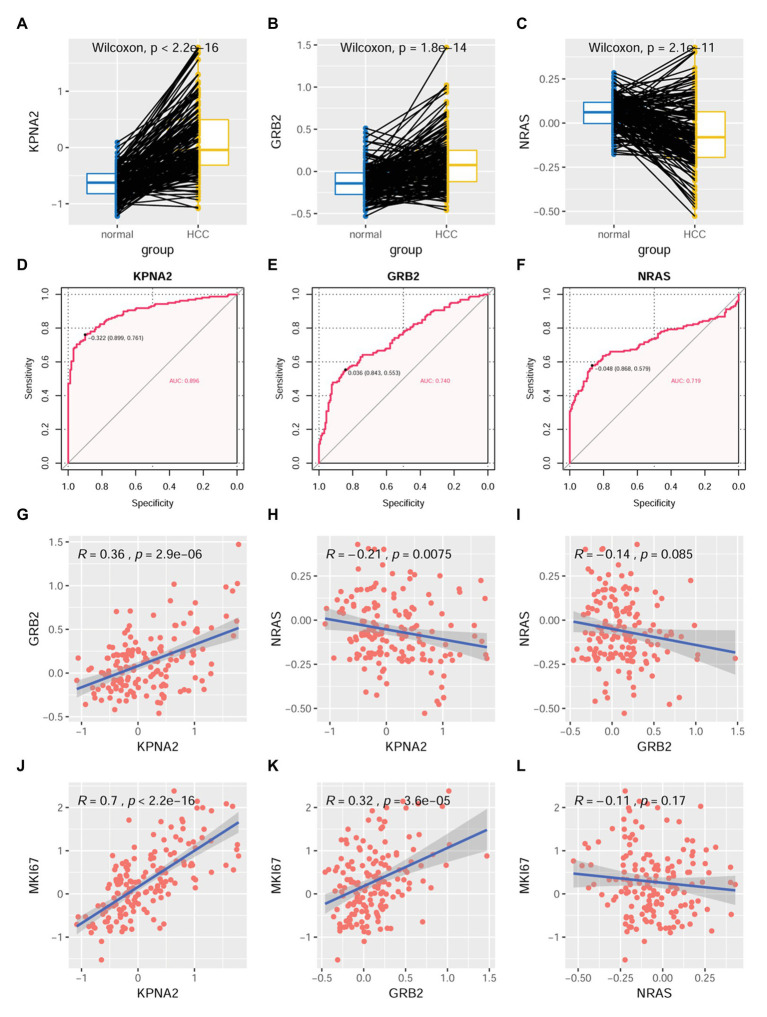
Dysregulations of KPNA2, GRB2, and NRAS proteins and their correlations with MKI67 in HCC. **(A)** Higher expression of KPNA2 in HCC than the normal liver tissues. **(B)** Higher expression of GRB2 in HCC than the normal liver tissues. **(C)** Lower expression of NRAS in HCC than the normal liver tissues. **(D–F)** ROC curve analysis of KPNA2, GRB2, and NRAS in discriminating HCC tumors from normal liver tissues, the cutoff point with biggest Youden index was labeled, and the corresponding specificity and sensitivity were shown. **(G)** Significant positive correlation between KPNA2 expression and GRB2 expression in HCC. **(H)** Significant negative correlation between KPNA2 expression and GRB2 expression in HCC. **(I)** There was no significant correlation between GRB2 expression and NRAS expression. **(J)** Significant positive correlation between KPNA2 expression and MKI67 expression. **(K)** Significant positive correlation between GRB2 expression and MKI67 expression. **(L)** There was no significant correlation between NRAS expression and MKI67 expression. AUC, area under the curve; ROC, receiver operating characteristic. For **(A–C)**, the *x*-axis and *y*-axis represented the sample groups and the relative expressions of the proteins (the protein abundance of the samples, with respect to the pooled reference sample, as log_2_ ratios), respectively. For **(D–F)**, the *x*-axis and *y*-axis represented the specificity and sensitivity of the variables in discriminating HCC tumors from the normal liver tissues. For **(G–L)**, the *x*-axis and *y*-axis represented the relative expressions of the variables (the protein abundance of the samples, with respect to the pooled reference sample, as log_2_ ratios). Paired-samples Wilcoxon test and Spearman correlation analysis were used for expressional comparison and correlation evaluation, respectively. For the analyses, *p* < 0.05 was considered significant.

At protein level, through CPTAC-HCC analysis, KPNA2 (*p* < 0.001; Figure 6A) and GRB2 (*p* < 0.001; Figure 6B) were found to be higher expressed in HCC tumors than their normal liver tissues, consistent with their upregulation in HCC tumors at mRNA level. However, in contrast to its overexpression in HCC tumors at mRNA level, NRAS protein was shown to be lower expressed in HCC tumors than their normal liver controls (*p* < 0.001; Figure 6C). Upon ROC curve analysis (Figures 6D–F), the diagnostic power of the three proteins in discriminating HCC from normal controls was shown with AUCs of 0.896, 0.740, and 0.719 for KPNA2, GRB2, and NRAS, respectively. At the optimal cutoff points, the sensitivity was 0.761, 0.553, and 0.579, and the specificity was 0.899, 0.843, and 0.868 for KPNA2, GRB2, and NRAS, respectively.

For their correlations, KPNA2 expression was presented to be positively correlated with GRB2 expression (*R* = 0.36, *p* < 0.001; Figure 6G) while negatively correlated with NRAS expression (*R* = −0.21, *p* < 0.01; Figure 6H). However, no significant expressional correlation between GRB2 and NRAS was shown (*p* > 0.05; Figure 6I).

**Figure 7:**
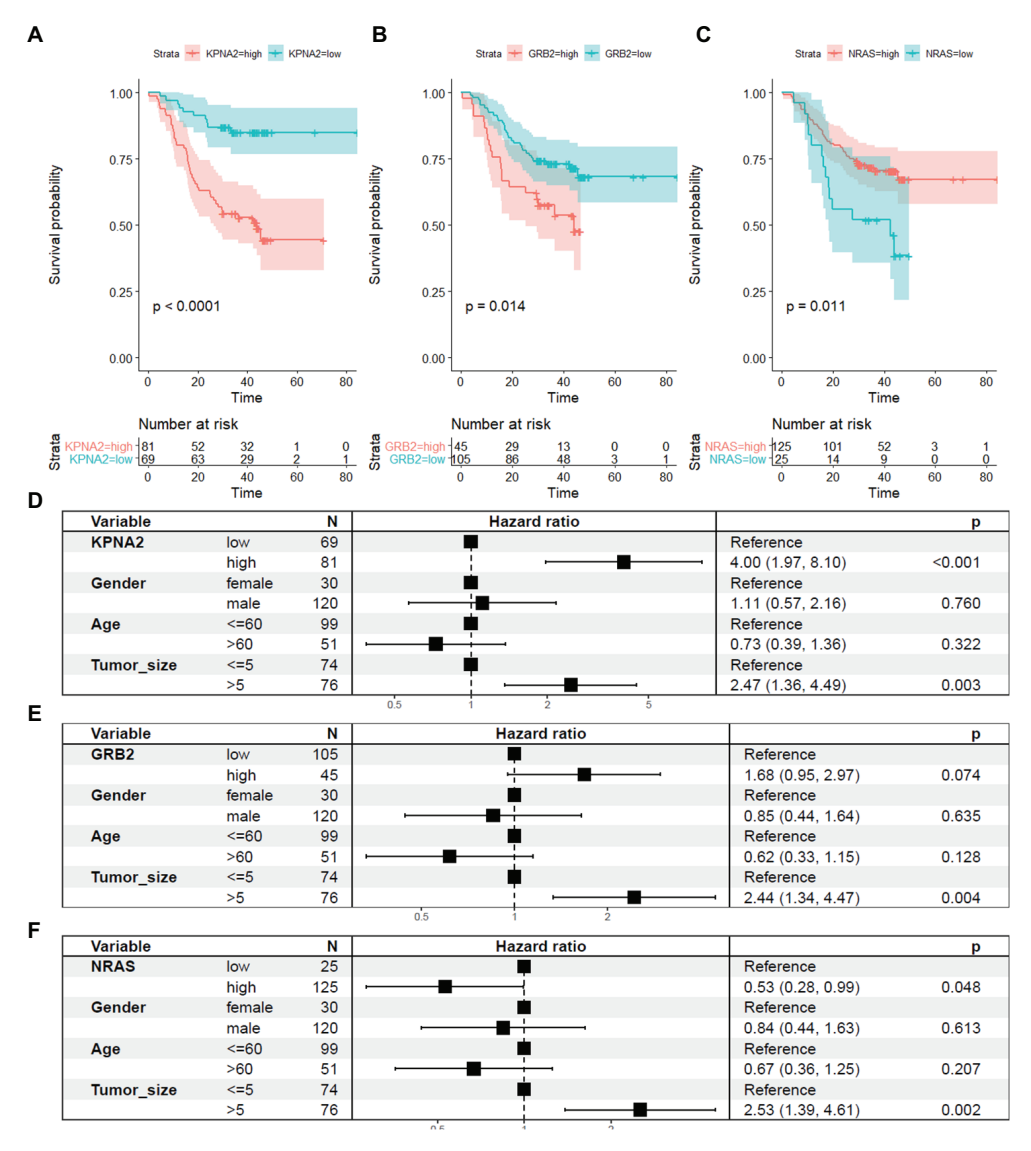
Prognostic roles of KPNA2, GRB2, and NRAS in HCC at protein level. **(A)** Higher expression of KPNA2 indicated shorter survival in HCC patients. **(B)** Higher expression of GRB2 indicated shorter survival in HCC patients. **(C)** Higher expression of NRAS indicated longer survival in HCC patients. **(D–F)** Prognostic effects of KPNA2, GRB2, and NRAS on HCC overall survival when adjusted for gender, age, and tumor size. Paired-samples Wilcoxon test was used for protein level comparisons. Kaplan-Meier survival analysis with log-rank test and multivariable Cox regression analysis were used for evaluation of the prognostic effects of the proteins. For all the analyses, *p* < 0.05 was considered statistically significant.

For their prognostic effects, through Kaplan-Meier analysis, at protein level, KPNA2 (*p* < 0.001; Figure 7A) and GRB2 (*p* < 0.05; Figure 7B) were shown to be unfavorable prognostic factors for HCC OS, consistent with their unfavorable prognostic effects at mRNA level. Interestingly, for NRAS (*p* < 0.05; Figure 7C), at protein level, it was shown to have favorable effects on HCC OS, inconsistent with its unfavorable effects on HCC OS. When adjusted for gender, age, and tumor size, the prognostic effects of KPNA2 (*p* < 0.001; Figure 7D) and NRAS (*p* < 0.05; Figure 7F) also existed, whereas the independent prognostic value of GRB2 was not so significant (*p* = 0.074, *p* > 0.05; Figure 7E).

### Associations of KPNA2, GRB2, and NRAS With HCC Proliferation

As shown in Figure 6, KPNA2 (*R* = 0.7, *p* < 0.001; Figure 6J) and GRB2 (*R* = 0.32, *p* < 0.001; Figure 6K) were shown to be significantly positively correlated with MKI67 expression, indicating their associations with HCC proliferation. Whereas no significant correlation was shown between NRAS expression and MKI67 expression in HCC (*p* > 0.05; Figure 6L).

### Prognostic Effects of RBPs of KPNA2, GRB2, and NRAS and Their Potential Roles in Dysregulations of the Three Proteins in HCC

**Figure 8:**
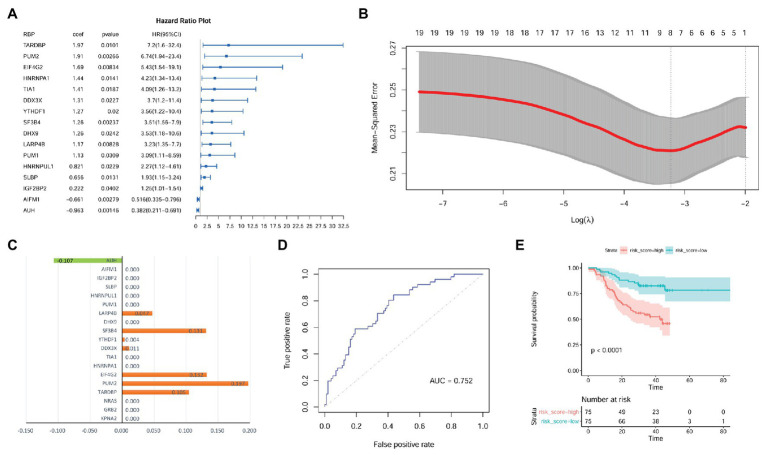
Identification of prognostic signature with RBPs of KPNA2, GRB2, and NRAS in HCC at protein level. **(A)** Gender, age, and tumor size corrected prognostic effects of RBPs of KPNA2, GRB2, and NRAS on HCC overall survival, and the right part was the visualization of the HR (95% CI). **(B)** Tuning parameter lambda (*λ*) selection using 10-fold cross validation. **(C)** Eight RBPs with absolute value of coefficient >0 were included in the LASSO regression model. The *x*-axis represented the coefficients of the RBPs, and the *y*-axis indicated the RBP variables. **(D)** The LASSO regression model could predicate the survival status with an AUC of 0.752 through ROC curve analysis. **(E)** HCC patients with higher risk score were indicated to have a shorter survival than those with lower risk score. RBPs, RNA-binding proteins; HR, hazard ratio; AUC, area under the curve; ROC, receiver operating characteristic. Ezcox package in R were used for Cox regression analysis of the RBPs **(A)** with the RBP expressions as covariates and age, gender, and tumor size as controls. Kaplan-Meier survival analysis with log-rank test **(E)** was used for the overall survival comparison between patients with high and low risk score, and the median value of the risk score was used as threshold for grouping the patients. For the analysis, *p* < 0.05 was considered significant.

As shown in Supplementary Table S3, there were 105, 112, and 114 RBPs that could bind to KPNA2, GRB2, and NRAS, respectively. As 94 of the RBPs were common for the three genes, there were 123 unique RBPs that could bind at least one of them. Because six RBPs were not available from the CPTAC-HCC dataset, only 117 RBPs (Supplementary Table S3) were investigated. Through multivariable Cox regression analysis (Supplementary Table S4), 16 RBPs were shown to have prognostic effects on HCC OS independent of gender, age, and tumor size in the CPTAC-HCC dataset (Figure 8A). Then, the 16 RBPs and KPNA2, GRB2, and NRAS were further applied to LASSO regression analysis, and an eight-RBP signature including AU RNA-binding methylglutaconyl-CoA hydratase (AUH), la ribonucleoprotein 4B (LARP4B), splicing factor 3b subunit 4 (SF3B4), YTH domain family 1 (YTHDF1), DEAD-box helicase 3 X-linked (DDX3X), eukaryotic translation initiation factor 4 gamma 2 (EIF4G2), pumilio RNA-binding family member 2 (PUM2), and TAR DNA-binding protein (TARDBP; Figures 8B,C) was identified. Through ROC curve analysis, the signature was shown to be able to predicate HCC survival status with an AUC of 0.752 (Figure 8D). With the model [risk score = (−0.107) ^*^ AUH_expression_ + 0.047 ^*^ LARP4B_expression_ + 0.131 ^*^ SF3B4_expression_ + 0.004 ^*^ YTHDF1_expression_ + 0.011 ^*^ DDX3X_expression_ + 0.132 ^*^ EIF4G2_expression_ + 0.197 ^*^ PUM2_expression_ + 0.105 * TARDBP_expression_], the risk scores of the HCC patients were evaluated, and through Kaplan-Meier survival analysis (Figure 8E), the patients with high risk score were shown to have a shorter OS than those with low risk score (*p* < 0.0001).

**Figure 9:**
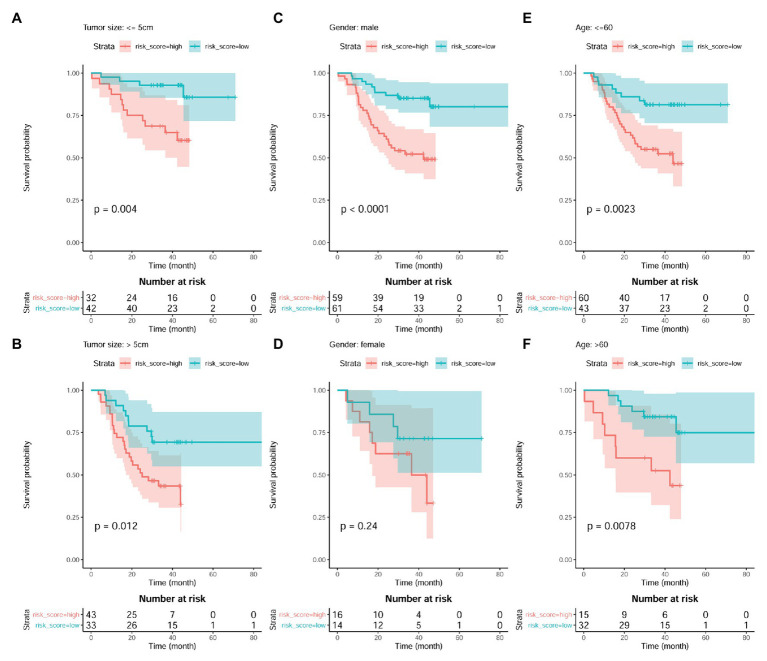
Prognostic effects of risk score in HCC patient of different gender, age, and tumor size groups. **(A,B)** Kaplan-Meier survival analysis of risk score in HCC patients with small tumors (tumor size ≤5 cm) group and large tumors (tumor size > 5 cm), respectively. **(C,D)** Kaplan-Meier survival analysis of risk score in male HCC patients and female ones, respectively. **(E,F)** Kaplan-Meier survival analysis of risk score in young HCC patients (≤60 years) and old ones (>60 years), respectively. Kaplan-Meier survival analysis with log-rank test was used, and *p* < 0.05 was considered as significant.

The independent prognostic value of the model was also investigated. As shown in Figures 9A,B, the prognostic effects of the model were shown in both HCC patients with smaller tumors (tumor size ≤ 5 cm; *p* = 0.004; Figure 9A) and those with larger ones (tumor size > 5 cm; *p* = 0.012; Figure 9B). In male patients, higher risk score also indicated shorter survival (*p* < 0.01; Figure 9C). Although the survival difference between female patients with high and low risk score was not so significant (*p* = 0.24), there was an obvious shorter survival trend in the patients with high risk score than those with low one (Figure 9D). In addition, comparing with the HCC patients with low risk score, the patients with high risk score were shown to survive shorter both in the younger group (*p* < 0.01; Figure 9E) and older group (*p* < 0.01; Figure 9F).

**Figure 10:**
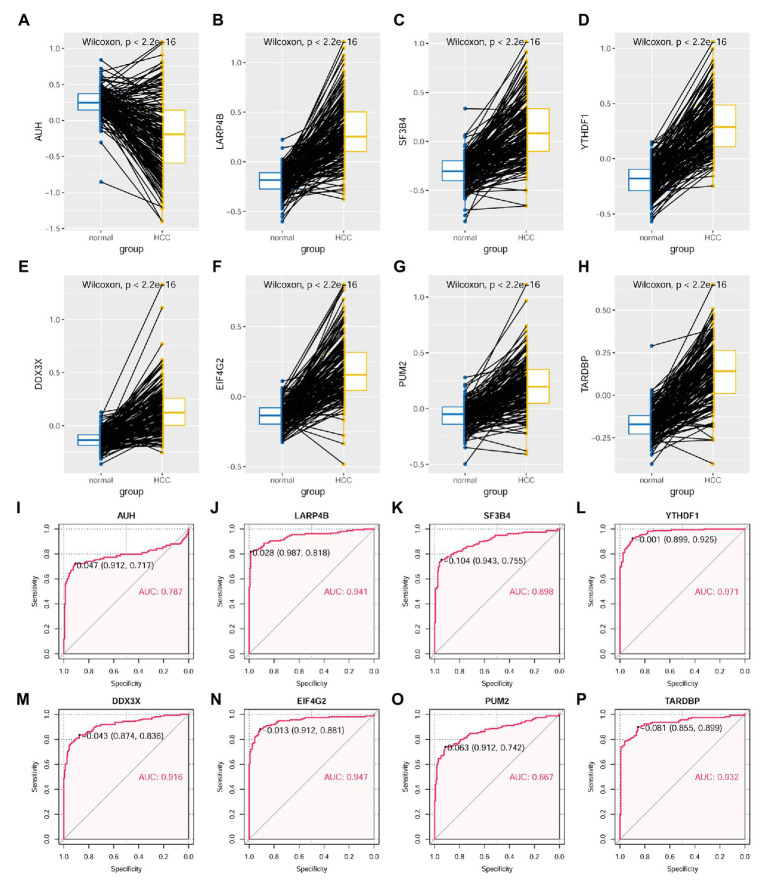
Expressional comparisons of RBPs of KPNA2, GRB2, and NRAS between HCC and normal liver tissues. **(A)** Lower expression of AUH in HCC tumors than their paired normal liver controls. **(B–H)** Higher expression of LARP4B, SF3B4, YTHDF1, DDX3X, EIF4G2, PUM2, and TARDBP in the HCC tumors than their paired normal liver controls. **(I–P)** ROC curve analysis of AUH, LARP4B, SF3B4, YTHDF1, DDX3X, EIF4G2, PUM2, and TARDBP in discriminating HCC tumors from normal liver tissues; the cutoff point with biggest Youden index was labeled, and the corresponding specificity and sensitivity were shown. AUC, area under the curve; ROC, receiver operating characteristic. For **(A–H)**, the *x*-axis and *y*-axis represented the sample groups and the protein abundance of the proteins in the samples, with respect to the pooled reference sample, as log_2_ ratios. For **(I–P)**, the *x*-axis and *y*-axis represented the specificity and sensitivity of the RBPs in discriminating HCC tumors from the normal liver tissues. Paired-samples Wilcoxon test and ROC analysis was used for expression comparisons of the protein levels and evaluation of the diagnostic power of the proteins, respectively. For all the analyses, *p* < 0.05 was considered significant.

As shown in Figure 10, through Wilcoxon test, the eight RBPs in the LASSO regression model above were also presented to be significantly differentially expressed between HCC tumors and their paired liver tissue controls. AUH, which was indicated to be a favorable prognostic factor for HCC OS, was shown to be downregulated (*p* < 0.01; Figure 10A), whereas the other seven unfavorable prognostic factors including LARP4B (*p* < 0.01; Figure 10B), SF3B4 (*p* < 0.01; Figure 10C), YTHDF1 (*p* < 0.01; Figure 10D), DDX3X (*p* < 0.01; Figure 10E), EIF4G2 (*p* < 0.01; Figure 10F), PUM2 (*p* < 0.01; Figure 10G), and TARDBP (*p* < 0.01; Figure 10H) were shown to be upregulated in HCC tumors. According to the ROC curve analysis (Figures 10I–P), all the eight RBPs could discriminate HCC tumors form normal livers with an AUC ranging from 0.787 (Figure 10I) to 0.971 (Figure 10L) individually. At the optimal cutoff points, their sensitivity ranged from 0.717 to 0.925, and the specificity ranged from 0.855 to 0.987, indicating their diagnostic potential in HCC diagnosis. Furthermore, all the eight RBPs were shown to be negatively or positively correlated with MKI67 expression in HCC tumors (*p* < 0.05, Supplementary Figure S3). Interestingly, the significant correlations of AUH (Supplementary Figure S3A), SF3B4 (Supplementary Figure S3C), DDX3X (Supplementary Figure S3E), PUM2 (Supplementary Figure S3G), and TARDBP (Supplementary Figure S3H) with MKI67 were only shown in the HCC tumors, while not in their paired normal liver tissues (*p* > 0.05), indicating the specific associations with HCC progression.

**Figure 11:**
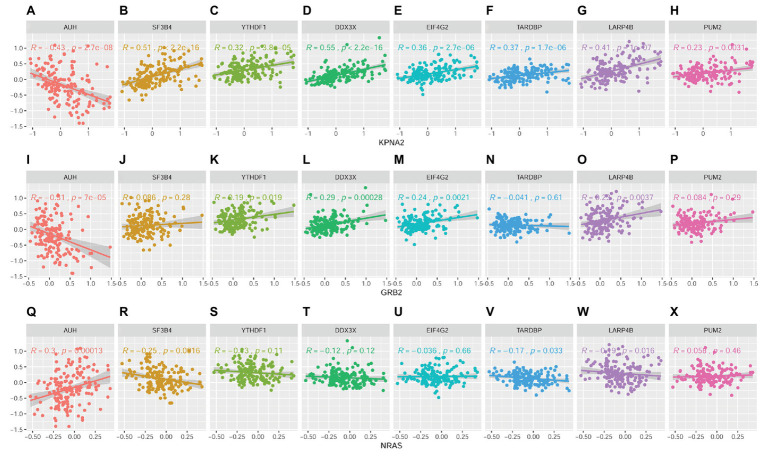
Correlations between RBPs and KPNA2, GRB2, and NRAS at protein level in HCC. **(A–H)** Correlations between KPNA2 and AUH, SF3B4, YTHDF1, DDX3X, EIF4G2, TARDBP, LARP4B, and PUM2, respectively. **(I–P)** Correlations between GRB2 and AUH, SF3B4, YTHDF1, DDX3X, EIF4G2, TARDBP, LARP4B, and PUM2, respectively. **(Q–X)** Correlations between NRAS and AUH, SF3B4, YTHDF1, DDX3X, EIF4G2, TARDBP, LARP4B, and PUM2, respectively. The *x*-axis and *y*-axis represented the abundance of the RBPs in the HCC samples, with respect to the pooled reference sample, as log_2_ ratios. Spearman correlation analysis was used, and *p* < 0.05 was considered significant.

Through Spearman correlation analysis (SupplementaryFigure S4 and Figure 11), the eight RBPs above were all indicated to be correlated with KPNA2 expression at protein level (*p* < 0.05; Figures 11A–H), and five (AUH, YTHDF1, DDX3X, EIF4G2, and LARP4B) and four (AUH, SF3B4, TARDBP, and LARP4B) of them were presented to be significantly correlated with GRB2 expression (*p* < 0.05; Figures 11I,K–M,O) and NRAS expression (*p* < 0.05; Figures 11Q,R,V,W), respectively. In addition, there were significant correlations between every two of the RBPs (Supplementary Figure S3), indicating their complicated associations. RBPs were implicated in RNA splicing, polyadenylation, mRNA stability, mRNA localization, and translation (Qin et al., 2020). Here, the dysregulations of RBPs might also contribute to the overexpression of KPNA2 and GRB2 and the underexpression of NRAS at protein level.

## Discussion

As a central immunomodulator of body, in liver, there are elements that can promote both immune tolerance and antitumor immunity, and the deregulation of the immunological network was demonstrated to be associated with chronic inflammation and tumor development (Protzer et al., 2012; Keenan et al., 2019). In a recent study, it was reported that in contrast to lung cancer and melanoma, HCC presented a low response rate to immune checkpoint inhibitors (Feng et al., 2020), indicating the complexity in the application of HCC immunotherapy. Because HCC occurs almost exclusively in the context of chronic inflammation (Ringelhan et al., 2018), further understanding of the associations of immune response would provide new clues for HCC diagnosis and therapy.

Although KPNA2 was implicated in many inflammatory processes (Liu et al., 2015; Pallett et al., 2019; Park et al., 2019), and its tumor-promoting activities in HCC (Gao et al., 2018; Lin et al., 2018; Zan et al., 2019) were shown in many studies, its associations with immune response in HCC and their clinical significance were not clearly illustrated. Here, we not only confirmed the upregulation and unfavorable prognostic effects of KPNA2 in HCC, and through further systemic analyses of its correlations with immune cell infiltrations in HCC tumors and normal liver tissues individually, we presented the specific positive correlation between KPNA2 expression and B-cell infiltration in the tumors. In addition, similar to KPNA2, in this study, B-cell infiltration was also shown to be an unfavorable prognostic factor for HCC OS, indicating the associations between B-cell infiltration and HCC progression.

BCR signaling pathway is crucial for B-cell response to the antigens, governing the processes of B-cell activation and fate decisions (Kwak et al., 2019). Here, we found the significant correlations of KPNA2 with many BCR signaling pathway genes including GRB2 and NRAS, which were shown to be dysregulated both at mRNA and protein levels. As a ubiquitously expressed adapter protein, GRB2 is crucial for normal development (Cheng et al., 1998) and implicated in cell proliferation (Downward, 1994). With its Src homology 2 (SH2) domain, GRB2 can also interact with several proteins in oncogenic signaling pathways including epidermal growth factor receptor, hepatocyte growth factor receptor, platelet-derived growth factor receptor, Bcr/Abl, and focal adhesion kinase (Lowenstein et al., 1992; Schlaepfer et al., 1994; Verbeek et al., 1997; Liu and McGlade, 1998; Fredericks and Ren, 2013). Recently, its tumor-promoting effects were shown in many malignancies including lung cancer (Yang et al., 2017a; Jiang et al., 2018; Mitra et al., 2018; Wang and Wang, 2020), gastric cancer (Ye et al., 2018), colorectal cancer (Ding et al., 2019), ovarian carcinoma (Huang et al., 2018), renal cell carcinoma (Gu et al., 2017), breast cancer (Lim et al., 2000; Haines et al., 2014; López-Cortés et al., 2020), and esophageal squamous cell carcinoma (Shi et al., 2018). In addition, in lung cancer (Chen et al., 2020), ovarian cancer (Xu et al., 2018), colorectal cancer (Agrawal et al., 2019), and breast cancer (Chen et al., 2018), its associations with the resistance of the tumors to chemotherapeutic drugs were presented, and its downregulation could reverse the resistant status or enhance the sensitivity to the drugs, indicating its potential as a chemotherapeutic target in the malignancies. Here, we presented the overexpression of GRB2, its positive correlation with cell proliferation marker MKI67, and its unfavorable prognostic roles in HCC, indicating its associations with HCC development and progression, consistent with the tumor-promoting activity of GRB2 in HCC reported in recent studies (Yang et al., 2018; Lv et al., 2020; Sun et al., 2020). Considering its positive correlation with KPNA2 and its important role in B-cell activation, we speculated they might play coordinate roles in HCC immunoregulation and progression. Besides its potential in HCC diagnosis and prognosis, GRB2 might be a target in HCC immunotherapy.

NRAS, a GTPase encoded by NRAS gene, originally identified in neuroblastoma cell lines, was considered as the third member of RAS family (Shimizu et al., 1983). Similar to the other two RAS members, KRAS and HRAS, NRAS was involved in cell growth, differentiation, and proliferation (Parker and Mattos, 2018; Murugan et al., 2019), and its deregulation was implicated in the metabolism of tumor cells, microenvironment remodeling, and the evasion of tumoral immune response (Mandalà et al., 2014). In fact, approximately 15–25% of all metastatic tumors were shown to have NRAS mutation (Boespflug et al., 2017), and the associations of NRAS with chemotherapy resistance were shown (Nguyen et al., 2020) in melanoma. In neuroblastoma, NRAS status was found to be associated with the sensitivity to SHP2 inhibitors (Valencia-Sama et al., 2020). In osteosarcoma, NRAS was reported to be associated with cell proliferation, migration, invasion, and methotrexate resistance (Li et al., 2020). Interestingly, in colorectal cancer, three NRAS mutants were shown to be associated with resistance to apoptosis, cytoskeletal reorganization, and loss of adhesion, whereas they have no effects on obvious effect on cell proliferation and motility (Yu and Garcia, 2020), indicating the complicated activities of the gene. In this study, we found the upregulation of NRAS and its unfavorable prognostic effects in HCC at mRNA level, consistent with a recent study that presented the overexpression of NRAS and its prognostic value in HCC (Dietrich et al., 2019). However, here, surprisingly, we found the downregulation of NRAS and its favorable prognostic effects in HCC at protein level, opposite to its mRNA level. As we included tumor and paired normal liver samples from 159 HCC patients for investigation at protein level here, the sample size is much larger than the previous study that included 46 HCC patients (Dietrich et al., 2019), and the results are reliable. In addition, considering the variety of regulatory factors implicated in the process of translation (Grafanaki et al., 2019; Moutinho et al., 2019; Kim et al., 2020), it is understandable to see the inconsistence between the mRNA and protein levels of specific genes. As no significant correlation of NRAS with MKI67 was shown at protein level in HCC, further studies were needed to investigate the specific functions of NRAS in HCC.

RBPs are implicated in various RNA processes including RNA splicing, RNA translation, and RNA degradation (Mohibi et al., 2019), and to some extent, they can regulate the fate of specific mRNAs (Zhang et al., 2018). In this study, through investigation of the RBPs of KPNA2, GRB2, and NRAS, we identified an eight-RBP signature that could predicate HCC OS effectively. Among the eight RBPs, AUH was shown to be downregulated and have favorable prognostic effects on HCC OS. Within the 3' untranslated region of lymphokines transcripts and some protooncogenes, there are many AU-rich elements that contain various numbers of reiterated AUUUA pentamers, and as *cis* elements, they serve as signal for rapid mRNA degradation (Chen et al., 1994). As an AU-binding protein, the downregulation of AUH in this study might lead to the decrease in the degradation of its binding mRNAs and contribute to the upregulation of corresponding mRNAs including KPNA2, GRB2, and NRAS. Noticeably, here, we presented an inconsistency of NRAS between its mRNA and protein levels. Besides its RNA-binding activity, as an enoyl-CoA hydratase, AUH also plays key roles in leucine degradation (IJlst et al., 2002; Ly et al., 2003) and mitochondrial protein synthesis (Richman et al., 2014). It was demonstrated that decrease or overexpression of the AUH protein in cells could lead to defects in mitochondrial translation. Here, NRAS was shown to be downregulated issn HCC at protein level, and there was a significant positive correlation between NRAS and AUH at protein level. We speculated that the decrease in AUH protein might be associated with the dysregulation of NRAS in HCC. However, considering the dual functions of AUH in RNA binding and protein translation and its involvement in the immune response to lipopolysaccharide (Zhang et al., 2020), further investigation is needed for its specific roles in NRAS dysregulation and HCC immunoregulation.

YTHDF1, one of the readers of N-6-methyladenosine (m6A) RNA methylation (Fu et al., 2014), could promote protein synthesis by interacting with translation machinery (Wang et al., 2015). Here, we presented the upregulation of YTHDC1 and its positive correlations with KPNA2 and GRB2 at protein level in HCC, indicating its associations with the dysregulation of the two proteins. In addition, as one element of the eight-PBP prognostic signature in HCC in this study, its unfavorable effects were also shown, consistent with previous studies (Huang et al., 2020; Wu et al., 2020). Through the correlation analysis, the significant correlations between the RBPs were shown, indicating their complicated associations and their coordinating roles during HCC progression.

In summary, B-cell infiltration was positively correlated with KPNA2 expression and unfavorably prognostic for HCC survival, indicating its associations with HCC progression. The significant correlations of KPNA2 with BCR signaling pathway indicated its association with BCR signaling. The dysregulation of BCR signaling pathway genes in HCC tumors indicated the crucial roles of BCR signaling pathway in the immune response of HCC. GRB2 and NRAS were dysregulated and had prognostic roles in HCC at both mRNA and protein levels, and they should be considered during HCC immunotherapy. Among the RBPs of KPNA2, GRB2, and NRAS, an eight-RBP signature with independent prognostic effects was identified. As all the eight RBPs were dysregulated in HCC, they might be new markers for HCC diagnosis and prognosis predication. Considering the complicated correlations between the RBPs, they might coordinate with each other in various biological processes, and their specific roles in the regulation of KPNA2, GRB2, and NRAS expressions needed further investigation.

## Data Availability Statement

All datasets presented in this study are included in the article/Supplementary Material.

## Author Contributions

XZ and LD conceived and designed the study. XZ, SF, JLZ, and FG collected and analyzed the data. XZ and JZZ interpreted the data. XZ drafted the manuscript. LD and SF reviewed and revised the manuscript. All authors contributed to the article and approved the submitted version.

### Conflict of Interest

The authors declare that the research was conducted in the absence of any commercial or financial relationships that could be construed as a potential conflict of interest.

## Footnotes

^1^https://CRAN.R-project.org/package=survminer

^2^https://www.genome.jp/kegg/

^3^https://CRAN.R-project.org/package=survminer

^4^http://starbase.sysu.edu.cn/index.php
