# Supplementary material for: KPNA2-Associated Immune Analyses Highlight the Dysregulation and Prognostic Effects of GRB2, NRAS, and Their RNA-Binding Proteins in Hepatocellular Carcinoma
Source: Front Genet. 2020 Oct 26;11:593273. doi: 10.3389/fgene.2020.593273 (PMC7649362; doi:10.3389/fgene.2020.593273)
Supplement: Supplementary file 1 [file Data_Sheet_1.pdf]

Table S1. The clinical features of HCC patients from Clinical Proteomic Tumor Analysis Consortium (CPTAC).

Variables	CPTAC-HCC (n=159)
Gender	n (%)
Male	128 (80.5%)
Female	31 (19.5%)
Age	n (%)
<=60	115(72.3%)
>60	44 (27.7%)
Tumor size (cm)	n (%)
<=5	77(48.4%)
>5	82 (51.6%)
Survival status	n (%)
Alive	99 (62.3%)
Dead	51 (32.1%)
NA	9 (5.6%)

NA, not available.

Table S2. Correlations between KPNA2 expression and BCR signaling pathway genes in HCC.

Gene	TCGA-HCC		ICGC-HCC	
	R	*p* value	*R*	*p* value
GRB2	0.428	0.000E+00**	0.563	0.000E+00**
PLCG2	-0.378	5.063E-14**	-0.414	1.783E-11**
NRAS	0.376	6.128E-14**	0.571	0.000E+00**
CD81	-0.351	3.174E-12**	-0.366	4.034E-09**
CHP1	-0.342	1.231E-11**	-0.142	0.027
PIK3R1	-0.333	4.969E-11**	-0.152	0.018
HRAS	0.320	2.933E-10	0.060	3.492E-01
NFKBIE	0.295	6.637E-09**	0.178	0.005**
NFKBIA	-0.293	8.831E-09**	-0.402	7.480E-11**
SOS2	-0.274	7.904E-08**	-0.020	0.755
PIK3R3	-0.266	1.887E-07**	0.004	0.945
PPP3CA	-0.265	2.106E-07**	-0.113	0.078
AKT1	-0.255	6.636E-07**	-0.346	3.096E-08**
AKT2	-0.245	1.787E-06**	-0.147	0.022
MAPK3	0.225	1.195E-05**	0.220	0.001**
FOS	-0.197	1.357E-04**	-0.239	1.677E-04**
CHP2	0.182	4.286E-04**	0.163	0.011
PPP3R1	-0.181	4.623E-04**	0.122	0.057
AKT3	-0.180	5.100E-04**	-0.185	0.004**
MAP2K2	0.177	6.219E-04**	0.061	0.342
IFITM1	-0.173	8.455E-04**	-0.295	2.791E-06**
BCL10	0.168	0.001**	0.224	4.213E-04**
NFKB1	-0.167	0.001**	0.019	0.774
RAC1	0.162	0.002**	0.073	0.256
NFATC2	0.161	0.002**	0.188	0.003**
BLNK	-0.154	0.003**	-0.287	5.449E-06**
PIK3R2	0.151	0.004**	0.373	1.955E-09**
RAC3	0.147	0.005	0.172	0.007**
NFAT5	-0.136	0.009	-0.144	0.025
SYK	0.135	0.009	0.099	0.123
PTPN6	0.133	0.010	-0.091	0.158
CD19	0.132	0.011	0.022	0.728
VAV2	-0.131	0.011	-0.122	0.058
MAP2K1	-0.129	0.013	0.030	0.639
JUN	-0.129	0.013	-0.151	0.019
RAC2	0.128	0.014	-0.065	0.315
NFATC3	-0.127	0.015	-0.335	9.063E-08**
CR2	0.122	0.019	0.116	0.072
PPP3CC	-0.119	0.022	-0.187	0.003**
NFATC1	-0.109	0.036	-0.149	0.020
RASGRP3	-0.100	0.054	-0.176	0.006**

CD22	-0.098	0.060	-0.135	0.036
PIK3CB	0.095	0.069	0.281	8.398E-06**
PIK3CA	-0.091	0.080	0.184	0.004**
LYN	0.085	0.103	0.155	0.016
MAPK1	0.074	0.154	0.371	2.446E-09**
PPP3R2	0.067	0.195	0.090	0.164
SOS1	-0.067	0.197	0.043	0.501
RAF1	0.067	0.199	0.205	0.001**
CD79B	-0.055	0.290	-0.019	0.768
LILRB3	0.055	0.291	-0.075	0.247
INPP5D	-0.053	0.310	-0.140	0.030
VAV1	0.053	0.313	-0.052	0.420
VAV3	-0.050	0.341	-0.033	0.611
BTK	0.048	0.360	-0.002	0.979
NFKBIB	0.045	0.391	-0.118	0.067
PIK3R5	-0.044	0.393	-0.147	0.022
MALT1	0.043	0.411	-0.178	0.005**
PIK3CG	-0.043	0.414	0.113	0.080
CD72	0.042	0.422	-0.060	0.351
PIK3CD	0.037	0.477	-0.104	0.105
KRAS	0.033	0.526	0.073	0.260
PIK3AP1	-0.033	0.528	-0.094	0.146
IKBKG	-0.025	0.629	-0.038	0.554
GSK3B	-0.025	0.631	0.063	0.325
CHUK	-0.023	0.658	0.267	2.508E-05**
NFATC4	0.021	0.682	-0.133	0.038
PRKCB	-0.020	0.701	0.065	0.312
CD79A	-0.020	0.708	-0.044	0.493
FCGR2B	0.019	0.710	-0.150	0.020
IKBKB	0.013	0.808	-0.346	3.068E-08**
RELA	0.009	0.859	-0.103	0.111
PPP3CB	0.009	0.865	0.070	0.274
CARD11	0.002	0.977	-0.180	0.005**
DAPP1	-0.001	0.978	-0.016	0.802

**, $|R| > 0.15$ and $p < 0.01$. Spearman correlation analysis was used and $|R| > 0.15$ with $p < 0.01$ was considered statistically significant.

Table S3. The RNA-binding proteins (RBPs)# of KPNA2, GRB2, and NRAS.

Serial number	Gene1	Gene2	Gene3	RBP
1	KPNA2	GRB2	NRAS	KHDRBS2
2	KPNA2	GRB2	NRAS	HNRNPUL1
3	KPNA2	GRB2	NRAS	SMNDC1
4	KPNA2	GRB2	NRAS	ELAVL1
5	KPNA2	GRB2	NRAS	FAM120A
6	KPNA2	GRB2	NRAS	ACIN1
7	KPNA2	GRB2	NRAS	SBDS
8	KPNA2	GRB2	NRAS	GNL3
9	KPNA2	GRB2	NRAS	SRSF3
10	KPNA2	GRB2	NRAS	RBM5
11	KPNA2	GRB2	NRAS	TAF15
12	KPNA2	GRB2	NRAS	AUH
13	KPNA2	GRB2	NRAS	DGCR8
14	KPNA2	GRB2	NRAS	CNBP
15	KPNA2	GRB2	NRAS	HNRNPA1
16	KPNA2	GRB2	NRAS	ZNF184
17	KPNA2	GRB2	NRAS	EIF4G2
18	KPNA2	GRB2	NRAS	NOP56
19	KPNA2	GRB2	NRAS	CPSF6
20	KPNA2	GRB2	NRAS	LIN28
21	KPNA2	GRB2	NRAS	LARP7
22	KPNA2	GRB2	NRAS	HNRNPA2B1
23	KPNA2	GRB2	NRAS	DDX42
24	KPNA2	GRB2	NRAS	PRPF8
25	KPNA2	GRB2	NRAS	TNRC6A
26	KPNA2	GRB2	NRAS	LSM11
27	KPNA2	GRB2	NRAS	SLTM
28	KPNA2	GRB2	NRAS	KHSRP
29	KPNA2	GRB2	NRAS	KHDRBS1
30	KPNA2	GRB2	NRAS	SRSF9
31	KPNA2	GRB2	NRAS	RNF219
32	KPNA2	GRB2	NRAS	HNRNPM
33	KPNA2	GRB2	NRAS	DDX54
34	KPNA2	GRB2	NRAS	SRSF1
35	KPNA2	GRB2	NRAS	FBL
36	KPNA2	GRB2	NRAS	TARDBP
37	KPNA2	GRB2	NRAS	U2AF1
38	KPNA2	GRB2	NRAS	TROVE2
39	KPNA2	GRB2	NRAS	MOV10
40	KPNA2	GRB2	NRAS	UPF1
41	KPNA2	GRB2	NRAS	NPM1
42	KPNA2	GRB2	NRAS	LIN28B

43	KPNA2	GRB2	NRAS	XRN2
44	KPNA2	GRB2	NRAS	PTBP1
45	KPNA2	GRB2	NRAS	FUS
46	KPNA2	GRB2	NRAS	IGF2BP3
47	KPNA2	GRB2	NRAS	NOP58
48	KPNA2	GRB2	NRAS	ADAR
49	KPNA2	GRB2	NRAS	TIA1
50	KPNA2	GRB2	NRAS	HNRNPD
51	KPNA2	GRB2	NRAS	RBM10
52	KPNA2	GRB2	NRAS	LIN28A
53	KPNA2	GRB2	NRAS	SF3A3
54	KPNA2	GRB2	NRAS	KHDRBS3
55	KPNA2	GRB2	NRAS	U2AF2
56	KPNA2	GRB2	NRAS	BUD13
57	KPNA2	GRB2	NRAS	CSTF2T
58	KPNA2	GRB2	NRAS	YWHAG
59	KPNA2	GRB2	NRAS	CELF2
60	KPNA2	GRB2	NRAS	IGF2BP1
61	KPNA2	GRB2	NRAS	HNRNPK
62	KPNA2	GRB2	NRAS	FXR2
63	KPNA2	GRB2	NRAS	DICER1
64	KPNA2	GRB2	NRAS	METTL3
65	KPNA2	GRB2	NRAS	EIF4G1
66	KPNA2	GRB2	NRAS	SND1
67	KPNA2	GRB2	NRAS	PCBP2
68	KPNA2	GRB2	NRAS	BCCIP
69	KPNA2	GRB2	NRAS	IGF2BP2
70	KPNA2	GRB2	NRAS	HNRNPC
71	KPNA2	GRB2	NRAS	TRA2A
72	KPNA2	GRB2	NRAS	RBFOX2
73	KPNA2	GRB2	NRAS	RBM47
74	KPNA2	GRB2	NRAS	GTF2F1
75	KPNA2	GRB2	NRAS	YTHDC1
76	KPNA2	GRB2	NRAS	SF3B4
77	KPNA2	GRB2	NRAS	SAFB2
78	KPNA2	GRB2	NRAS	YTHDF1
79	KPNA2	GRB2	NRAS	FXR1
80	KPNA2	GRB2	NRAS	TIAL1
81	KPNA2	GRB2	NRAS	EIF4A3
82	KPNA2	GRB2	NRAS	ILF3
83	KPNA2	GRB2	NRAS	SRSF10
84	KPNA2	GRB2	NRAS	RANGAP1
85	KPNA2	GRB2	NRAS	RBM27
86	KPNA2	GRB2	NRAS	RBM22

87	KPNA2	GRB2	NRAS	FMR1
88	KPNA2	GRB2	NRAS	SRSF7
89	KPNA2	GRB2	NRAS	DDX3X
90	KPNA2	GRB2	NRAS	NONO
91	KPNA2	GRB2	NRAS	MSI2
92	KPNA2	GRB2	NRAS	NUMA1
93	KPNA2	GRB2	NRAS	QKI
94	KPNA2	GRB2	NRAS	HNRNPU
95	KPNA2		NRAS	SLBP
96	KPNA2		NRAS	CBX7
97	KPNA2		NRAS	PUM1
98	KPNA2	GRB2		AIFM1
99	KPNA2	GRB2		FTO
100	KPNA2	GRB2		DKC1
101	KPNA2	GRB2		VIM
102	KPNA2	GRB2		DHX9
103	KPNA2	GRB2		HNRNPL
104	KPNA2			METTL14
105	KPNA2			RBM6
106		GRB2	NRAS	TARBP2
107		GRB2	NRAS	EWSR1
108		GRB2	NRAS	ZFP36
109		GRB2	NRAS	CAPRIN1
110		GRB2	NRAS	LARP4B
111		GRB2	NRAS	ZC3H7B
112		GRB2	NRAS	ELAVL3
113		GRB2	NRAS	MSI1
114		GRB2	NRAS	RTCB
115		GRB2	NRAS	PUM2
116		GRB2	NRAS	RC3H1
117			NRAS	NCBP3
118			NRAS	EIF3G
119			NRAS	EIF3A
120			NRAS	WTAP
121			NRAS	EIF3D
122			NRAS	ALYREF
123		GRB2		MBNL2

[#] As the data of KHDRBS2, LIN28, LSM11, LIN28A, ZFP36 and ELAVL3 were not available, only 117 RBPs were investigated in this study. RBP, RNA-binding protein.

Table S4. The gender-, age- and tumor size- corrected prognostic effects of the RBPs on HCC overall survival.

RBP	beta	HR (95%CI)	P value
AUH	-0.963	0.382(0.211-0.691)	0.00146**
SF3B4	1.26	3.51(1.56-7.9)	0.00237**
PUM2	1.91	6.74(1.94-23.4)	0.00266**
AIFM1	-0.661	0.516(0.335-0.796)	0.00279**
LARP4B	1.17	3.23(1.35-7.7)	0.00828**
EIF4G2	1.69	5.43(1.54-19.1)	0.00834**
TARDBP	1.97	7.2(1.6-32.4)	0.0101*
SLBP	0.656	1.93(1.15-3.24)	0.0131*
HNRNPA1	1.44	4.23(1.34-13.4)	0.0141*
TIA1	1.41	4.09(1.26-13.2)	0.0187*
YTHDF1	1.27	3.56(1.22-10.4)	0.02*
DDX3X	1.31	3.7(1.2-11.4)	0.0227*
HNRNPUL1	0.821	2.27(1.12-4.61)	0.0229*
DHX9	1.26	3.53(1.18-10.6)	0.0242*
PUM1	1.13	3.09(1.11-8.59)	0.0309*
IGF2BP2	0.222	1.25(1.01-1.54)	0.0402*
SF3A3	1.12	3.06(0.993-9.45)	0.0514
MSI1	0.449	1.57(0.997-2.46)	0.0517
FUS	0.59	1.8(0.994-3.27)	0.0522
U2AF1	0.798	2.22(0.968-5.1)	0.0596
CPSF6	1.11	3.02(0.896-10.2)	0.0745
KHDRBS3	0.779	2.18(0.908-5.24)	0.0812
U2AF2	1.05	2.86(0.868-9.41)	0.084
HNRNPL	1.17	3.21(0.831-12.4)	0.0906
XRN2	0.989	2.69(0.855-8.45)	0.0907
CAPRIN1	0.884	2.42(0.866-6.76)	0.0918
MSI2	0.551	1.73(0.907-3.32)	0.0962
NONO	1.01	2.74(0.835-8.99)	0.0964
SND1	-1.04	0.354(0.102-1.23)	0.103
TIAL1	1.13	3.11(0.765-12.6)	0.113
HNRNPU	0.75	2.12(0.831-5.4)	0.116
HNRNPM	1.04	2.84(0.753-10.7)	0.123
RBM47	-0.713	0.49(0.195-1.23)	0.129
ILF3	0.731	2.08(0.788-5.47)	0.139
VIM	0.513	1.67(0.846-3.3)	0.14
EIF4A3	1	2.73(0.7-10.6)	0.148
ALYREF	0.723	2.06(0.758-5.61)	0.157
ADAR	0.559	1.75(0.779-3.92)	0.176
SAFB2	0.64	1.9(0.748-4.81)	0.178
IGF2BP3	0.121	1.13(0.942-1.35)	0.19
DICER1	0.447	1.56(0.799-3.06)	0.192

SRSF1	0.843	2.32(0.634-8.51)	0.203
RANGAP1	0.532	1.7(0.747-3.87)	0.205
SRSF3	1.03	2.8(0.564-13.9)	0.208
KHDRBS1	0.669	1.95(0.669-5.69)	0.221
SRSF10	0.743	2.1(0.637-6.93)	0.223
RNF219	0.313	1.37(0.823-2.27)	0.226
METTL3	-0.538	0.584(0.242-1.41)	0.232
METTL14	-0.592	0.553(0.208-1.47)	0.236
SMNDC1	0.429	1.54(0.744-3.17)	0.246
HNRNPD	0.606	1.83(0.645-5.21)	0.256
FXR1	0.504	1.66(0.694-3.95)	0.256
CSTF2T	0.626	1.87(0.615-5.68)	0.27
RBM22	0.599	1.82(0.627-5.29)	0.271
RTCB	-0.786	0.456(0.113-1.84)	0.271
HNRNPC	0.778	2.18(0.528-8.97)	0.282
KHSRP	0.544	1.72(0.63-4.71)	0.29
TNRC6A	-0.388	0.679(0.326-1.41)	0.299
DGCR8	0.483	1.62(0.645-4.08)	0.304
TARBP2	0.392	1.48(0.689-3.18)	0.314
PRPF8	0.661	1.94(0.52-7.22)	0.325
GNL3	0.316	1.37(0.724-2.6)	0.333
TRA2A	0.529	1.7(0.576-5.01)	0.337
GTF2F1	-0.421	0.656(0.277-1.55)	0.338
RC3H1	0.349	1.42(0.689-2.92)	0.343
CBX7	-0.289	0.749(0.411-1.36)	0.345
CELF2	0.493	1.64(0.572-4.69)	0.358
LARP7	-0.559	0.572(0.17-1.93)	0.367
HNRNPA2B1	0.593	1.81(0.498-6.58)	0.368
PCBP2	0.612	1.84(0.476-7.15)	0.376
TROVE2	0.398	1.49(0.607-3.65)	0.385
SRSF9	0.269	1.31(0.704-2.43)	0.396
FMR1	0.348	1.42(0.634-3.16)	0.396
DKC1	0.331	1.39(0.614-3.16)	0.428
FAM120A	-0.463	0.63(0.199-2)	0.432
NOP56	0.287	1.33(0.618-2.87)	0.464
ZC3H7B	-0.532	0.587(0.141-2.45)	0.466
FXR2	0.373	1.45(0.531-3.97)	0.467
EIF4G1	-0.394	0.674(0.219-2.07)	0.491
BCCIP	0.246	1.28(0.63-2.59)	0.496
SRSF7	0.553	1.74(0.346-8.72)	0.502
YTHDC1	0.346	1.41(0.511-3.91)	0.505
LIN28B	-0.0498	0.951(0.818-1.11)	0.518
ELAVL1	0.362	1.44(0.473-4.36)	0.523
TAF15	-0.311	0.733(0.274-1.96)	0.536

PTBP1	0.362	1.44(0.414-4.98)	0.568
EIF3D	0.425	1.53(0.329-7.12)	0.587
EIF3A	0.399	1.49(0.335-6.64)	0.6
NCBP3	0.233	1.26(0.521-3.06)	0.606
ZNF184	0.182	1.2(0.593-2.43)	0.612
IGF2BP1	0.0434	1.04(0.88-1.24)	0.619
HNRNPK	0.378	1.46(0.327-6.5)	0.62
NPM1	0.171	1.19(0.569-2.47)	0.648
EWSR1	-0.262	0.77(0.245-2.42)	0.654
SBDS	0.233	1.26(0.453-3.52)	0.655
DDX54	-0.177	0.838(0.35-2.01)	0.692
QKI	-0.18	0.835(0.332-2.1)	0.702
WTAP	0.178	1.19(0.474-3.01)	0.706
BUD13	-0.155	0.857(0.365-2.01)	0.723
FBL	0.139	1.15(0.53-2.49)	0.725
RBFOX2	0.271	1.31(0.273-6.3)	0.735
RBM5	0.23	1.26(0.329-4.82)	0.738
NOP58	0.123	1.13(0.529-2.42)	0.751
MOV10	0.0966	1.1(0.578-2.1)	0.769
EIF3G	0.204	1.23(0.295-5.1)	0.778
UPF1	0.157	1.17(0.374-3.66)	0.787
ACIN1	0.154	1.17(0.359-3.8)	0.798
DDX42	0.105	1.11(0.36-3.43)	0.855
YWHAG	-0.155	0.856(0.158-4.64)	0.857
RBM6	0.0857	1.09(0.372-3.19)	0.876
FTO	0.0575	1.06(0.486-2.31)	0.885
MBNL2	0.0564	1.06(0.462-2.42)	0.894
CNBP	-0.0485	0.953(0.464-1.96)	0.895
RBM27	-0.052	0.949(0.354-2.55)	0.918
NUMA1	-0.0225	0.978(0.467-2.05)	0.952
RBM10	0.0254	1.03(0.331-3.18)	0.965
SLTM	0.0106	1.01(0.33-3.09)	0.985

*, $p < 0.05$; **, $p < 0.01$. Multivariable cox regression analysis was used and $p < 0.05$ was considered statistically significant.

Figure S1

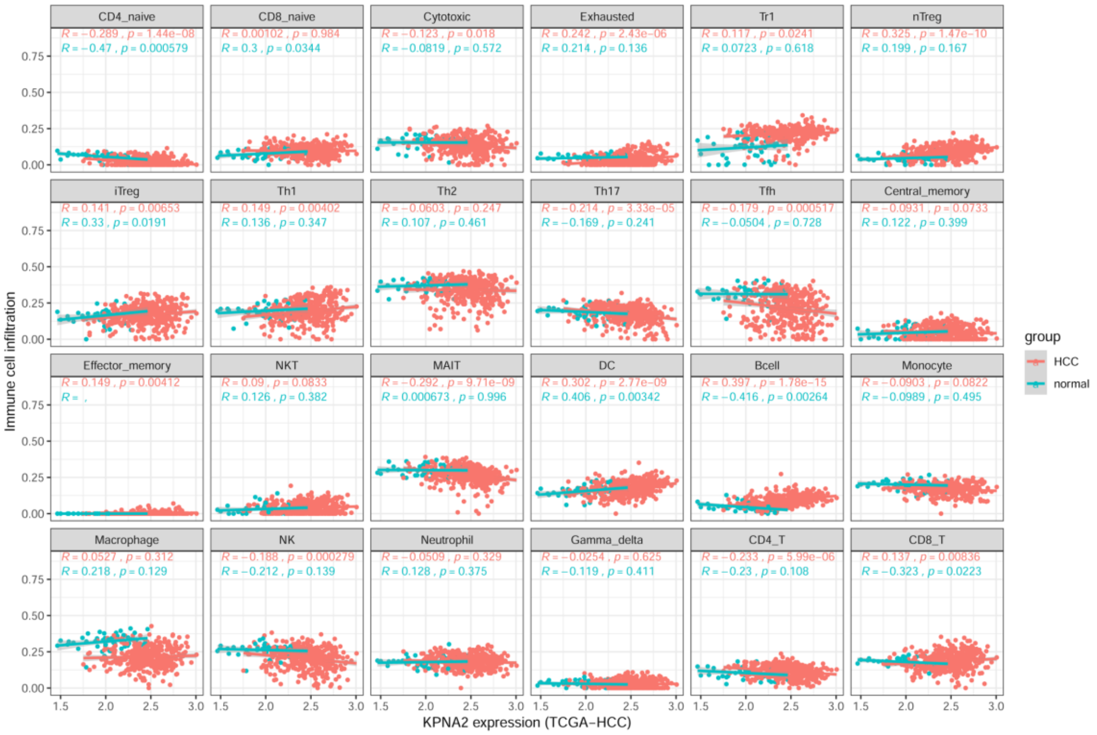

FIGURE S1. Correlations between KPNA2 expression and immune infiltrations in the samples of TCGA-HCC dataset. Spearman correlation analysis was used and $|R| > 0.15$ with p <0.01 was considered statistically significant. R, correlation coefficient.

Figure S2

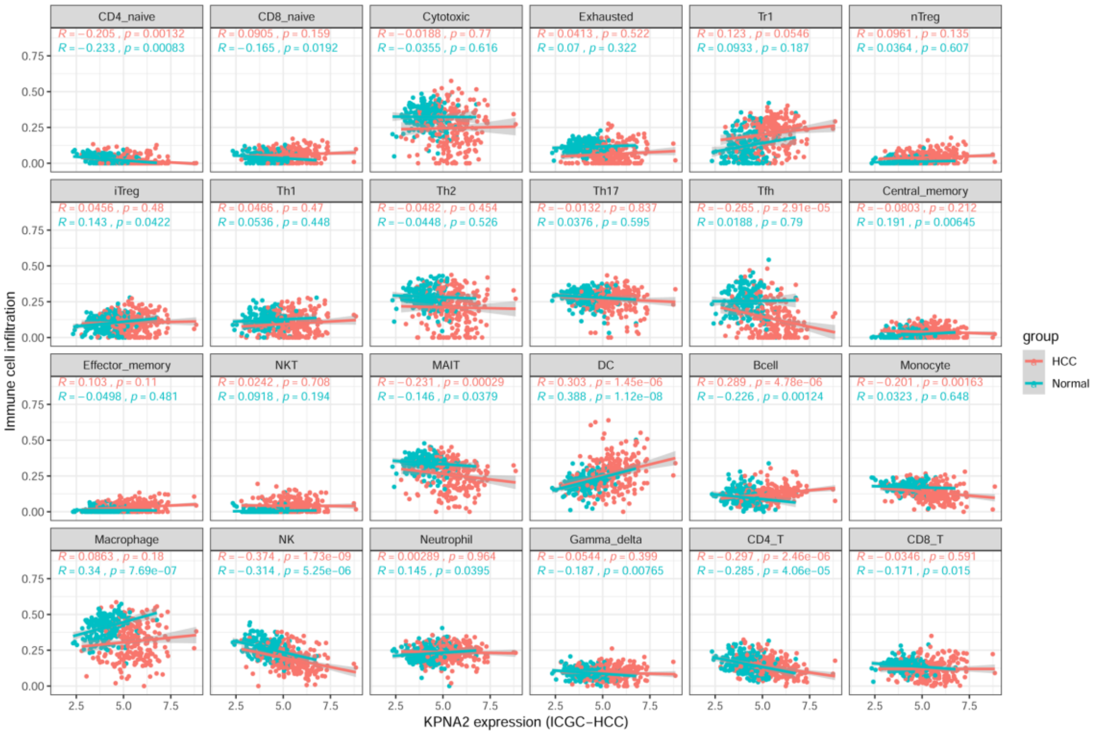

FIGURE S2. Correlations between KPNA2 expression and immune infiltrations in the samples of ICGC-HCC dataset. Spearman correlation analysis was used and $|R| > 0.15$ with $p < 0.01$ was considered statistically significant. *R*, correlation coefficient.

Figure S3

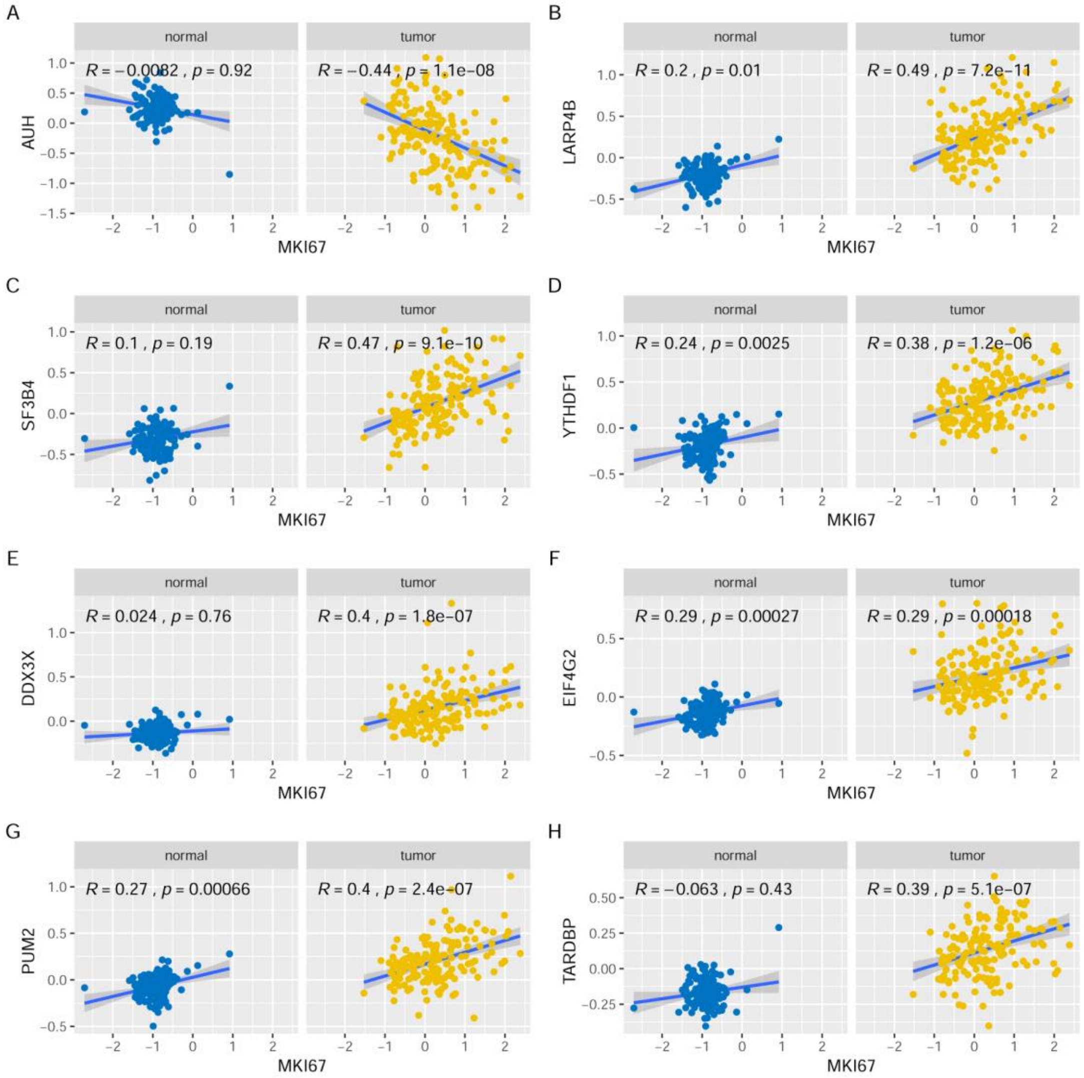

Figure S3. Correlations of RBPs with MKI67 in HCC tumors and normal liver tissues. Spearman correlation analysis was used and $p < 0.05$ was considered significant.

Figure S4

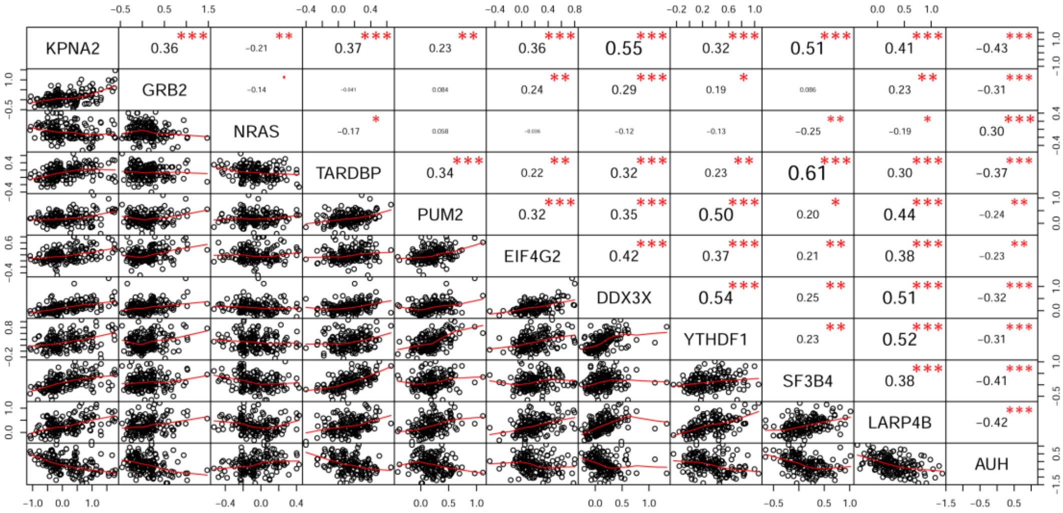

Figure S3. Correlations of KPNA2, GRB2, NRAS, and their RBPs in HCC at protein level. $^{\cdot}$, $0.05 < p < 0.10$; *, $p < 0.05$; **, $p < 0.01$. Spearman correlation analysis was used and $p < 0.05$ was considered significant.
